# Quercetin Inhibits LPS-Induced Inflammation and ox-LDL-Induced Lipid Deposition

**DOI:** 10.3389/fphar.2017.00040

**Published:** 2017-02-03

**Authors:** Feng Xue, Xiaobo Nie, Jianping Shi, Qingxue Liu, Ziwei Wang, Xiting Li, Jinqiu Zhou, Jia Su, Mingming Xue, Wei-Dong Chen, Yan-Dong Wang

**Affiliations:** ^1^Key Laboratory of Molecular Pathology, School of Basic Medical Science, Inner Mongolia Medical UniversityHohhot, China; ^2^Key Laboratory of Receptors-Mediated Gene Regulation and Drug Discovery, School of Medicine, Henan UniversityKaifeng, China; ^3^Chinese Internal Medicine Teaching and Researching Section, Inner Mongolia Medical UniversityHohhot, China; ^4^State Key Laboratory of Chemical Resource Engineering, College of Life Science and Technology, Beijing University of Chemical TechnologyBeijing, China; ^5^Internal Medicine Section, No. 253 Hospital of PLAHohhot, China

**Keywords:** quercetin (QCT), atherosclerosis (AS), inflammation, lipid deposition, Mongolian medicine

## Abstract

Aberrant activation of inflammation and excess accumulation of lipids play crucial role in the occurrence and progression of atherosclerosis (AS). Quercetin (QCT) has been tested effectively to cure AS. It is widely distributed in plant foods and has been proved to have potential antioxidative and anticancer activities. However, the underlying molecular mechanisms of OCT in AS are not completely understood. In the present study, we stimulated murine RAW264.7 cells with lipopolysaccharide (LPS) or oxidized low-density lipoproteins (ox-LDL) to mimic the development of AS. The data show that QCT treatment leads to an obvious decrease of multiple inflammatory cytokines in transcript level, including interleukin (IL)-1α, IL-1β, IL-2, IL-10, macrophage chemoattractant protein-1 (MCP-1), and cyclooxygenase-2 (COX-2) induced by LPS. Moreover, expressions of other factors that contribute to the AS development, such as matrix metalloproteinase-1 (MMP-1) and suppressor of cytokine signaling 3 (SOCS3) induced by LPS are also downregulated by QCT. Furthermore, we found that QCT suppressed LPS-induced the phosphorylation of STAT3. Meanwhile, QCT could ameliorate lipid deposition and overproduction of reactive oxygen species induced by ox-LDL, and block the expression of lectin-like oxidized LDL receptor-1 (LOX-1) in cultured macrophages. Taken together, our data reveal that QCT has obvious anti-inflammatory and antioxidant virtues and could be a therapeutic agent for the prevention and treatment of AS.

## Introduction

Cardiovascular disease (CVD) is the leading cause of morbidity and mortality worldwide (Jiang et al., 2013), with more than 16 million death annually due to its complications. Atherosclerosis (AS) is responsible for a large proportion of cardiovascular related mortality (Liu et al., 2016). As a complex and chronic progressive inflammatory disease, AS is characterized by the abnormal accumulation of fibrous elements and lipids in large and medium-sized arteries. AS is frequently asymptomatic for several decades until the occurrence of severe cardiovascular disorders such as stroke or heart attack (Bale et al., 2016).

Inflammation is involved in every stage of AS, ranging from injury in vessel endothelial cells to the rupture of plaque in the end. In series of cellular events for the progression of AS, formation of lipid-laden macrophages (foam cells) plays a vital role in response to the inflammation-associated stimuli (Angelovich et al., 2015). Typically, its formation is associated with a state of hyperlipidaemia and abnormal accumulation of ox-LDL. A broad range of inflammatory cytokines, such as interleukin (IL)-1 and IL-2 produced by macrophages will exert inflammatory effects and accelerate the progression of AS (Signorelli et al., 2014; Eaton et al., 2015). Furthermore, multiple evidences suggested that chronic inflammation could be an independent risk factor to promote the atherogenesis. In particular, the activation of STAT3, as a prominent modulator of inflammation during AS, coordinates a platform for synergistic amplification leading to pro-atherogenic responses (Szelag et al., 2016). In addition, continued proinflammatory events could activate macrophages to produce excessive reactive oxygen species (ROS), which induces the apoptosis of foam cells and is involved in the subsequent plaque formation in AS lesion progression (Ekstrand et al., 2015; Manea et al., 2015). Besides, imbalance of cholesterol influx, synthesis and eﬄux could disrupt the cholesterol homeostasis and contribute the generation of foam cells (Chistiakov et al., 2015). Therefore, inhibiting the generation of inflammation and ox-LDL-induced lipid deposition in macrophage and the modulation of intracellular ROS levels may be attractive therapeutic strategies for preventing AS (Back and Hansson, 2015).

As the natural medicine, Mongolian medicine has been proven to treat CVDs effectively in clinical practice, with the total efficiency over 90% (Meng, 2003; Jin and Chen, 2013; Shi, 2015). However, its development has been seriously hampered due to the lack of complete theoretical system and experimental researches. Therefore, it is very meaningful to explore detailed molecular mechanisms to improve their therapeutic management in AS. Quercetin (QCT) is a crucial ingredient in Mongolian prescription Amin Erden (Shi, 2015). It has to be mentioned that Amin Erden, as a commercialized prescription of traditional Mongolian medicine, has been used to treat CVDs and AS in China (Meng, 2003; Jin and Chen, 2013; Shi, 2015). Several studies have demonstrated QCT markedly inhibited the development of hypertension in hypertension mouse model and the antihypertensive effects of QCT may be due to enhanced eNOS activity and decreased NADPH oxidase-mediated superoxide anion (O2) generation associated with reduced p47 expression (Duarte et al., 2002; Sanchez et al., 2006). The antioxidant property of QCT has also been mentioned frequently on account of reducing the susceptibility of ox-LDL and its aggregation in early stages of AS (Aviram et al., 1997). In addition, QCT protects against oxidative damage *in vitro* and *in vivo* through suppressing ROS generation (Abengozar-Vela et al., 2015; Hu et al., 2015; Sharma et al., 2015; Ben Salem et al., 2016). QCT has been reported to antagonize STAT3 signaling in B-cell lymphoma and breast cancer cells (Li et al., 2014; Seo et al., 2016) and down-regulate IL-6/STAT3 signaling in lung cancer cells (Mukherjee and Khuda-Bukhsh, 2015).

In the current study, we identify QCT as a negative regulator of LPS-induced inflammation at least partly through suppressing STAT3 signaling in macrophages. Furthermore, it is found that OCT suppressed ox-LDL-induced lipid deposition and ROS production. These findings suggest that QCT could be a therapeutic agent for prevention and treatment of AS.

## Materials and Methods

### Reagents

Quercetin, LPS (from *Escherichia coli* 0111:B4), Dimethyl Sulfoxide (DMSO), 2′,7′-dichlorofluorescin diacetate (DCFH-DA) and STAT3 inhibitor S3I-201 were purchased from Sigma Chemical (St. Louis, MO, USA). Ox-LDL was purchased from Shanghai Qcbio Science &Technologies Co., Ltd. QCT was dissolved in DMSO (≥ 99.9%) and added into the medium directly (the final work concentration of DMSO is 0.1%).

### Macrophage Cell Line Culture

The murine RAW264.7 cell line was purchased from School of Basic Medicine of Peking Union Medical College (Beijing, China). Cells were cultured in Dulbecco’s Modified Eagle’s Medium (2 mM glutamine, 1 mM pyruvate, 4.5 g/l glucose) supplemented with 10% heat-inactivated fetal bovine serum (FBS) (Gibco, USA) and antibiotics (100 U/ml penicillin and streptomycin) (Gibco, USA) at 37°C in a humidified atmosphere of 5% CO_2_ (Wang et al., 2011). To evaluate the anti-atherogenic effect of QCT, cells were randomly divided into three groups in the following ways: control (vehicle DMSO); LPS/ox-LDL treated; LPS/ox-LDL+QCT treated. Each group had a minimum of three replicated wells. After 24 h of incubation, cells were subjected to QCT at a final concentration of 20 μM for 24 h (Jing et al., 2016), followed by treatment with LPS (500 ng/ml) for another 6 h, or treated with ox-LDL (50 μg/ml) for an additional 24 h. Subsequently, cells were harvested and extracted for further analysis.

### RNA Extraction and Quantitative Real Time PCR (qRT-PCR)

See the supporting information (Wang et al., 2008). Sequences of the primers used for real-time PCR are given in Supplementary Table S1.

### Oil Red O Staining

RAW264.7 cells were divided into three groups [control (vehicle DMSO), ox-LDL treated, ox-LDL+QCT treated] as described above. Cells were pretreated with QCT (20 μM) or vehicle DMSO for 24 h followed with/without ox-LDL (20 μg/ml) treatment for another 24 h. Cell cultures were rinsed once with phosphate-buffered saline (PBS) and fixed with 10% (v/v) formaldehyde for 15 min at room temperature. After washing three times with double-distilled water (ddH_2_O), the cells were incubated with filtered Oil Red O solution at room temperature for another 15 min, followed by further washing five times with ddH_2_O to remove the background staining. Cells from each culture well were observed and photographed randomly under the microscope (TE200; Nikon, Tokyo, Japan).

### Cholesterol Quantitation Assay

To explore the effect of QCT on cholesterol accumulation in macrophages, murine RAW264.7 cells were treated with ox-LDL (20 μg/ml) and QCT (20 μM) as the method described above. Cells were collected and processed for cholesterol extraction (Cholesterol Quantitation Kit from Sigma-Aldrich, MAK043) according to the instruction (Kain et al., 2015). Total or free cholesterol concentration of samples was estimated using commercial cholesterol quantitation kit (Sigma-Aldrich, USA) by the automated analyzer according to the instruction and previous report. Briefly, 50 μl of the diluted standards and samples were incubated with 50 μl of reaction mixes containing cholesterol assay buffer, cholesterol probe, cholesterol enzyme mix, cholesterol esterase for 60 min at 37°C. After incubation, the absorbance was measured at 570 nm on a multiscan spectrum (EnSpire Multimode Plate Readers, PerkinElmer) and the cholesterol standard curve (0, 20, 40, 60, 80, 100 μg/ml) was generated using the standard cholesterol solutions. All standards and samples were run in duplicate.

### Detection of ROS Production

To examine whether the production of ROS induced by ox-LDL treatment could be blocked by QCT in macrophages, RAW264.7 cells were subjected to the treatment of QCT (20 μM) for 24 h followed by ox-LDL (50 μg/ml) treatment for another 24 h. ROS production was detected as described previously (Wang et al., 2015). As non-fluorescent DCFH-DA could be de-esterified intracellularly and turns into fluorescent 2′,7′-dichlorofluorescin (DCFH) upon oxidation by free radicals, cells pellets were washed with prechilled PBS and processed for DCFH-DA treatment for 60 min at 37°C. Subsequently, ROS production in response to oxidation in macrophages was detected by spectrofluorimetry, which exciting light at 488 nm and emitting light at 525 nm.

### Protein Extraction and Immunoblot Detection of Phosphorylated STAT3

See the supporting information. Beta-actin was used as a loading control.

### Statistical Analysis

Error bar for the experiments represents the standard deviation of the mean value (mean value ± SD) from at least three independent experiments. All statistical analyses were carried out using the SPSS.11 software (IBM, USA). The Student’s *t*-test was used to calculate *P*-values between two groups. For comparisons between multiple groups, a one-way ANOVA analysis was performed. *P*-values of ≤0.05 were considered as statistically significant.

## Results

### QCT Inhibits LPS-Induced Inflammatory Gene Expression in RAW264.7 Cells

**FIGURE 1:**
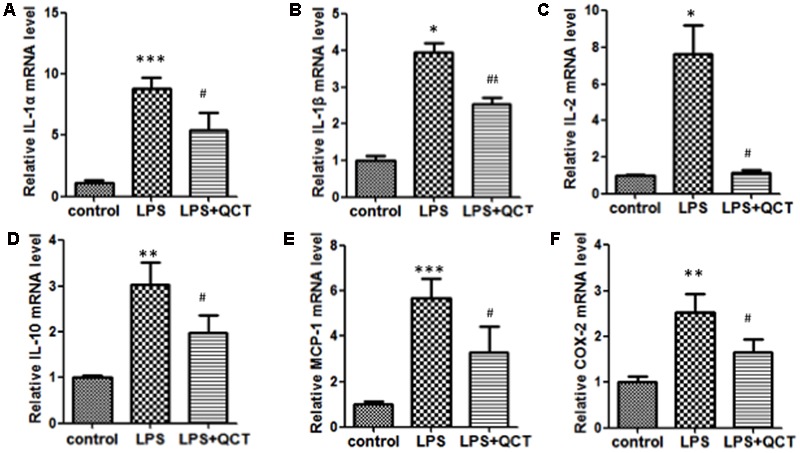
**Quercetin (QCT) inhibited inflammation in LPS-treated RAW264.7 cells.** RAW 264.7 cells were grown in six-well plates for 24 h and treated with 20 μM QCT for 24 h, and then stimulated with 500 ng/ml of LPS for another 6 h. The cells were harvested and transcript levels of the following cytokines were examined by qRT-PCR. Relative mRNA levels were shown in **(A)** IL-1α, **(B)** IL-1β, **(C)** IL-2, **(D)** IL-10, **(E)** MCP-1, and **(F)** COX-2.^∗^*P* < 0.05, ^∗∗^*P* < 0.01, ^∗∗∗^*P* < 0.001 vs. the control group; ^#^*P* < 0.05, ^##^*P* < 0.01 vs. the LPS-treated group. One-way ANOVA analysis was used to calculate *P*-values. The bars represent mean ± SD. All data represent at least three independent experiments.

Accumulation of vascular inflammation and immune cells is an important nongenetic factor of AS. To determine whether QCT suppressed inflammation response, we firstly investigated the effects of QCT alone on non-stimulated cells. The results showed that QCT alone decreased mRNA levels of IL-1α, IL-1β, IL-10, COX-2, SOCS3, and LOX-1 (Supplementary Figure S1). Moreover, we used LPS to induce inflammation in RAW 264.7 cells. As shown in **Figures 1A–F**, activation of macrophages by LPS resulted in the increase of mRNA levels of proinflammatory genes, such as IL-1α, IL-1β, IL-2, IL-10, MCP-1, and COX-2, compared with the vehicle control group. In contrast, treatment with QCT obviously attenuated the LPS-induced mRNA expression of IL-1α, IL-1β, IL-2, IL-10, MCP-1, and COX-2. Some of the results were also confirmed using Enzyme-Linked Immunosorbent Assay (ELISA) assay. It revealed that QCT suppressed IL-1β, MCP-1 and COX-2 protein expression in RAW264.7 Cells (Supplementary Figure S2). In addition, the effective treatment concentration of QCT used in this study did not cause cytotoxic effect based on our MTT assay (Supplementary Figure S3). We also determined the effects of QCT with different concentrations on cytokine expression triggered by LPS. It was found that QCT suppressed gene expression of IL-1β, MCP-1 and COX-2 in a dose-dependent manner (Supplementary Figure S4A). Taken together, these data manifested that QCT might be an effective anti-inflammatory agent.

### QCT Treatment Inhibits LPS-Induced Activation of STAT3 in RAW 264.7 Cells

**FIGURE 2:**
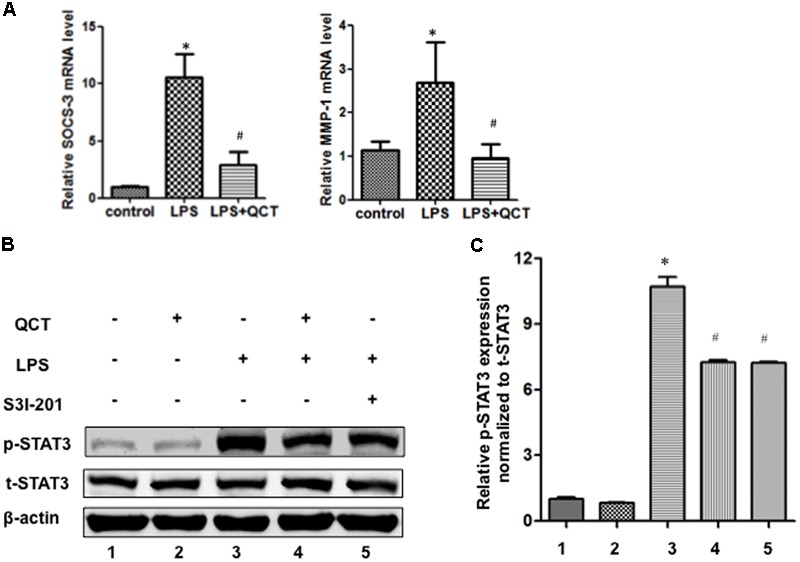
**Quercetin treatment inhibits LPS-induced activation of STAT3 in RAW264.7 cells. (A)** QCT suppresses SOCS3 and MMP-1 expression induced by LPS. RAW264.7 cells were grown in six-well plates for 24 h and treated with 20 μM QCT for 24 h, and then stimulated with 500 ng/ml of LPS for another 6 h. The cells were harvested and transcript levels of the following cytokines were examined by qRT-PCR. ^∗^*P* < 0.05 vs. the control group; ^#^*P* < 0.05 vs. the LPS-treated group. The bars represent mean ± SD. **(B)** Representative immunoblot showing phosphorylated STAT3 (p-STAT3) and total STAT3 (t-STAT3) protein levels in RAW264.7 cells treated with DMSO, QCT (20 μM), LPS (500 ng/ml), LPS (500 ng/ml) + QCT (20 μM), and LPS (500 ng/ml) + S3I-201 (100 μM). β-actin as a loading control. **(C)** Densitometry was used to quantify relative p-STAT3 protein levels normalized to t-STAT3 protein levels ^∗^*P* < 0.05 vs. control group; ^#^*P* < 0.05 vs. LPS-induced group. One-way ANOVA analysis was used to calculate *P*-values. The bars represent mean ± SD. All data represent at least three independent experiments.

The activation of STAT3 signal pathway has been reported to be involved in controlling many key inflammatory genes identified in AS. In the present work, to evaluate the underlying mechanism by which QCT acts to suppress the LPS-induced AS inflammation, we tested whether QCT antagonized STAT3 signaling pathway. As shown in **Figure 2A**, we found QCT suppresses the mRNA levels of two STAT3 target genes SOCS3 and MMP-1 induced by LPS. IL-6 is a specific inducer for STAT3 signaling. We found that IL-6 treatment alleviated the suppression of QCT on LPS-induced mRNA levels of IL-1α, IL-6, SOCS3, and iNOS (Supplementary Figure S5A). Furthermore, we used antibody against IL-6 to test the role of IL-6 in this process. We found that anti-IL-6 antibody enhanced the effects of QCT on gene expression induced by LPS (Supplementary Figure S5B). S3I-201, a specific inhibitor of STAT3 activity, has been used to demonstrate a direct link between QCT-induced inhibition of the STAT3 pathway and other biological parameters. We found mRNA levels of some proinflammatory genes in LPS+QCT group was similar to that of LPS+S3I-201 group (Supplementary Figure S6). Prostaglandin E2 (PGE2) is an inducer of STAT3. Inhibition of PGE2 synthesis has been an important anti-inflammatory strategy (Ramanan et al., 2016). We found QCT suppressed IL-6-induced PGE2 production in RAW264.7 cells (Supplementary Figure S7). These results suggest that QCT could inhibit inflammatory gene expression at least partially through suppressing STAT3 pathway.

The phosphorylation of STAT3 in RAW264.7 cells treated with LPS was significantly increased as compared to control groups (**Figures 2B,C**). By contrast, QCT treatment significantly suppressed STAT3 phosphorylation induced by LPS (**Figures 2B,C**). S3I-201 was used for negative control. Taken together, it suggests that QCT may exert an anti-inflammatory effect on AS through inhibiting STAT3 pathway. We also tested the effects of QCT on AKT and IκBα signaling pathways. We found that QCT suppressed the phosphorylation of AKT induced by LPS (Supplementary Figure S8) but not the phosphorylation of IκBα (data not shown).

### QCT Ameliorates ox-LDL-Induced Inflammation in RAW264.7 Cells

**FIGURE 3:**
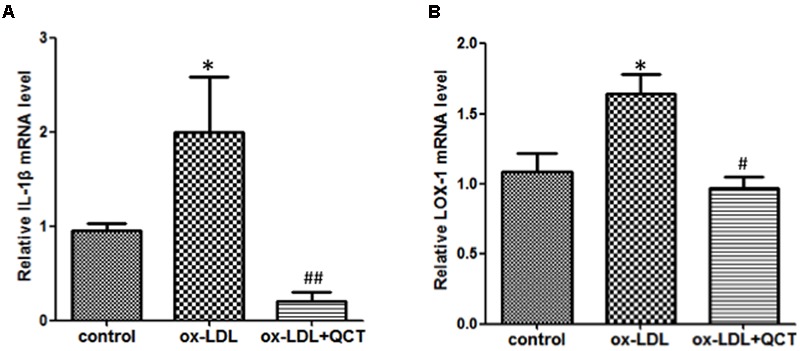
**Quercetin ameliorated oxidized low-density lipoproteins (ox-LDL)-induced inflammation in RAW264.7 cells.** RAW264.7 cells growing in six-well plates were pre-incubated with 20 μM of QCT for 24 h before treatment with 50 μg/ml of ox-LDL. Cells were harvested and transcript levels of IL-1β **(A)** and LOX-1 **(B)** were measured by qRT-PCR. ^∗^*P* < 0.05 vs. the control group; ^#^*P* < 0.05 and ^##^*P* < 0.01 vs. ox-LDL-induced group. One-way ANOVA analysis was used to calculate *P*-values. The bars represent mean ± SD. All data represent at least three independent experiments.

Abundant evidences have confirmed that uptake of ox-LDL by macrophages to form foam cells is another main determinant for the endothelial dysfunction and development of AS. In order to determine the anti-inflammation properties of QCT, we pretreated RAW264.7 cells with QCT followed by stimulated with ox-LDL. Compared with the control group, activation of macrophages by ox-LDL leaded to the mRNA level increase of proinflammatory gene IL-1β (**Figure 3A**) and induced the transcription of LOX-1 (**Figure 3B**), which is the specific receptor of ox-LDL. QCT treatment decreased the gene expression of IL-1β (**Figure 3A**) and LOX-1 (**Figure 3B**) induced by ox-LDL. We also determined the effects of QCT with different concentrations on IL-1β and LOX-1 expression triggered by ox-LDL. It was found that IL-1β and LOX-1 gene expression was suppressed by QCT in a dose-dependent manner (Supplementary Figure S4B).

### QCT Suppresses ox-LDL-Induced Lipid Deposition in RAW264.7 Cells

**FIGURE 4:**
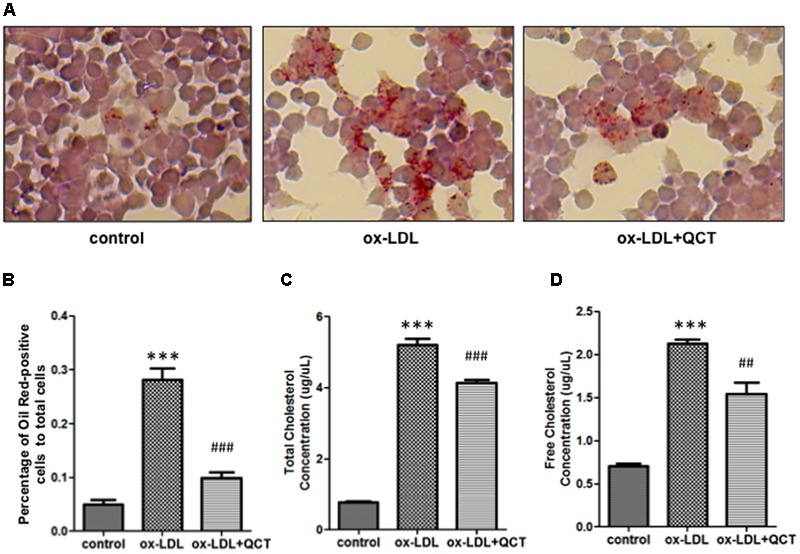
**Quercetin decreased ox-LDL-induced lipid deposition in RAW264.7 cells. (A)** Murine RAW264.7 cells treated with ox-LDL (20 μg/ml) and QCT (20 μM) were fixed with formaldehyde for 15 min followed by stained with Oil Red O solution for an additional 15 min and then photographed randomly. Representative photographs of Oil Red O stained cells in different groups were shown. **(B)** The percentage of Oil Red-positive cells to total cells was calculated using Image-Pro Plus Image Analysis Software (Media Cybernetics, USA). **(C)** and **(D)** RAW264.7 cells were treated with ox-LDL and QCT as the method described above. Total cholesterol concentrations **(C)** and free cholesterol concentrations **(D)** of cells in different groups were quantitatively analyzed by using cholesterol quantitation kit. ^∗∗∗^*P* < 0.001 vs. the control group; ^##^*P* < 0.01, ^###^*P* < 0.001 vs. ox-LDL-induced group. One-way ANOVA analysis was used to calculate *P*-values. The bars represent mean ± SD. All data represent at least three independent experiments.

Lipid accumulation in macrophages and the formation of foam cells are crucial steps to promote the development of AS. To investigate the effects of the QCT on ox-LDL-induced lipid deposition, RAW264.7 cells were pretreated with QCT for 24 h and then exposed to ox-LDL for another 24 h, followed by stained with Oil Red O. Microscopic examination showed that ox-LDL exposure increased the cytoplasmic lipid accumulation (number of fat droplets) in macrophages compared with that in control cells and supplementation of QCT markedly decreased ox-LDL-induced the lipid levels through decreasing the percentage of Oil Red-positive cells (**Figures 4A,B**). QCT treatment did not cause the decrease in the number of cells in Oil Red staining results (Supplementary Figure S9). Therefore, the Oil Red O staining results indicate the inhibitory effects of QCT on cellular lipid accumulation.

The generation of foam cells is related to the imbalance of cholesterol influx, esterification and eﬄux. We detected obvious increase of free cholesterol and total cholesterol accumulation in ox-LDL-treated macrophages through biochemical analysis. In contrast, QCT treatment induced significant reduction of total cholesterol and free cholesterol concentration in ox-LDL-treated cells, respectively (**Figures 4C,D**).

### QCT Abrogates ox-LDL-Induced Overproduction of ROS in RAW 264.7 Cells

**FIGURE 5:**
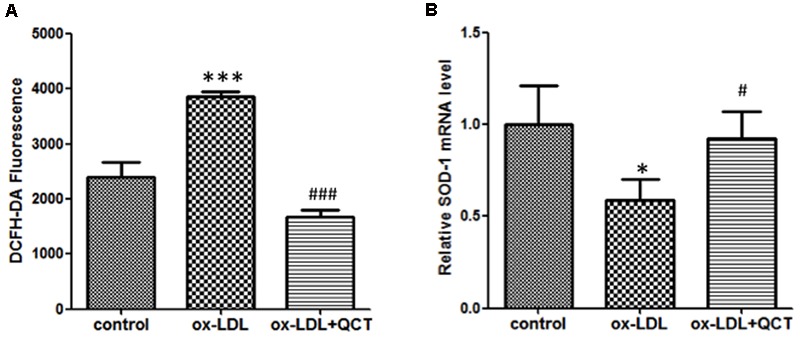
**Quercetin abrogated ox-LDL-induced overproduction of reactive oxygen species (ROS) in RAW264.7 cells. (A)** Murine RAW264.7 cells treated with ox-LDL (50 μg/ml) and QCT (20 μM) were processed for DCFH-DA treatment for 60 min. ROS production in response to oxidation and the subsequent treatment of QCT in RAW264.7 cells was detected by spectrofluorimetry and quantified. **(B)** The expression of SOD-1 in RAW264.7 cells from different groups was detected by qRT-PCR. ^∗^*P* < 0.05, ^∗∗∗^*P* < 0.001 vs. control group; ^#^*P* < 0.05, ^###^*P* < 0.001 vs. ox-LDL-treated group. One-way ANOVA analysis was used to calculate *P*-values. The bars represent mean ± SD. All data represent at least three independent experiments.

Previous studies have proved that phagocytosis of ox-LDL by macrophages gave rise to the activation of macrophage and increased production of ROS, finally contributing to atherosclerotic lesion progression. Therefore, we investigated the effect of QCT on the generation of ROS. We found that 24 h incubation with ox-LDL resulted in a remarkable increase of ROS production (**Figure 5A**). QCT could significantly abrogate the ox-LDL-induced overproduction of ROS. These results were also confirmed using the OxiSelect ROS Assay Kit (Cell Biolabs Inc.) (data not shown). Moreover, ox-LDL treatment diminished the expression of SOD-1 transcript (**Figure 5B**). QCT increased the SOD-1 levels reduced by ox-LDL in macrophages (**Figure 5B**), indicating a significant antioxidant capacity of QCT.

## Discussion

Quercetin has been reported to decrease cytokine expression *in vitro* and *in vivo* through antagonizing NF-κB and other signaling pathways (Zhang et al., 2016; Zou et al., 2016). It is reported that QCT also has antitumor functions through suppressing STAT3 signaling (Li et al., 2014; Seo et al., 2016). In addition, QCT protects against oxidative damage *in vitro* and *in vivo* through suppressing ROS generation (Abengozar-Vela et al., 2015; Hu et al., 2015; Sharma et al., 2015; Ben Salem et al., 2016). In the current work, we show that QCT reduced levels of inflammatory factors, induced by LPS through blocking STAT-3 pathway in RAW264.7 cells. We demonstrated that QCT is able to inhibit LPS-induced inflammation and ox-LDL-induced lipid deposition, which suggest that QCT could be a therapeutic agent for the prevention and treatment of AS. In addition, QCT is one of the main active constituents of the Mongolian prescription “Amin Erden.” All data in the current work are indirect molecular evidences to prove the effectiveness of Amin Erden on AS, though more experiments should be carried out in the future work.

Oxidized low-density lipoproteins has been demonstrated to be crucial for the pathogenesis of AS and ox-LDL exposure to endothelial cells or macrophages could be considered as a simple model of AS *in vitro*. As the main ox-LDL receptor, LOX-1 has been confirmed to be closely involved in AS pathogenesis. Overexpression of LOX-1 induces intramyocardial vasculopathy in hyperlipidemic mice and the effect is probably mediated through the endothelial dysfunction induced by LOX-1 (Inoue et al., 2005). Conversely, deletion of LOX-1 could reduce the AS in LDL receptor deficient mice fed with high cholesterol diet (Mehta et al., 2007). In this study, we investigated the role of LOX-1 in an ox-LDL induced AS model and found a protective effect of QCT. It suggests that the decreased accumulation of lipid observed in macrophages treated with QCT could be mediated by the declining level of LOX-1.

Previous reports suggest that STAT3 controls the inflammatory cascade more extensively in CVDs. Inflammation in AS is partially due to the enhancement of STAT3 signaling. In vascular smooth muscle cells (VSMCs) and macrophages, STAT3 has been confirmed to regulate the transcription of abundant number of cytokines (Sahar et al., 2005). Moreover, decreasing the phosphorylation of STAT3 suppressed both inflammation and monocyte-to-macrophage differentiation (Vasamsetti et al., 2015). In the current work, we found QCT treatment suppressed the phosphorylation of STAT3 induced by LPS, which was similar to the published observations for other natural flavonoids (Wiejak et al., 2013). It indicates that QCT suppresses the inflammation in AS through inhibiting the STAT3 pathway.

## Conclusion

Treatment of QCT, as an important ingredient in Mongolian medicine Amin Erden, could effectively suppress inflammation response stimulated by LPS through inhibiting the phosphorylation of STAT3. QCT reduces the lipid deposition and ROS production induced by ox-LDL in RAW 264.7 cells. Taken together, it is inspiring to demonstrate that QCT has clinical value for the prevention and treatment of AS, and this Mongolian prescription is worth to be promoted for anti-AS therapy.

## Author Contributions

Y-DW, W-DC, and MX conceived and designed the study. FX, XN, JPS, JZ, JS, XL, QL, and ZW performed experiments. Y-DW, W-DC, FX, XN, and JPS analyzed data. FX, XN, and JPS wrote the paper. Y-DW, W-DC, and MX reviewed and edited the manuscript. All authors read and approved the manuscript.

## Conflict of Interest Statement

The authors declare that the research was conducted in the absence of any commercial or financial relationships that could be construed as a potential conflict of interest.
