# Supplementary material for: Quercetin Inhibits LPS-Induced Inflammation and ox-LDL-Induced Lipid Deposition
Source: Front Pharmacol. 2017 Feb 3;8:40. doi: 10.3389/fphar.2017.00040 (PMC5289956; doi:10.3389/fphar.2017.00040)
Supplement: Supplementary file 1 [file Presentation_1.PDF]

Quercetin inhibits LPS-induced inflammation and oxLDL-induced lipid deposition

Feng Xue,[1,#] Xiaobo Nie,[2,#] Jianping Shi,[3,#] Qingxue Liu,[1] Ziwei Wang,[4] Xiting Li,[2] Jinqiu Zhou,[5] Jia Su,[4] Mingming Xue,[1,*] Wei-Dong Chen,[1,2,*] Yan-Dong Wang[4,*]

[#] Feng Xue, Xiaobo Nie and Jianping Shi contributed equally to this work.

[1] Key Laboratory of Molecular Pathology, School of Basic Medical Science, Inner Mongolia Medical University, Hohhot, Inner Mongolia, P.R. China,

[2] Key Laboratory of Receptors-Mediated Gene Regulation and Drug Discovery, School of Medicine, Henan University, Kaifeng, Henan 475004, P.R. China

[3] Chinese Internal Medicine Teaching and Researching Section, Inner Mongolia Medical University, Hohhot, Inner Mongolia, P.R. China

[4] State Key Laboratory of Chemical Resource Engineering, College of Life Science and Technology, Beijing University of Chemical Technology, Beijing, P. R. China

[5] Internal Medicine Section, No. 253 Hospital of PLA, Hohhot, Inner Mongolia, P.R. China

Table 1 The sequences of the primers used in qRT-PCR

Primer name	Sequences
SOD-1(F)	CCAGTGCAGG ACCTCATTTT
SOD-1(R)	GGCCAATGAT GGAATGCTCT
LOX-1(F)	GCTGCTATGA CTCTGGTCAT
LOX-1(R)	TACGATCCTG CTGAGTAAGG
IL-1β(F)	CTCATTGTGG CTGTGGAGAA
IL-1β(R)	CACACACCAG CAGGTTATCA
SOCS3(F)	TTCTTCACGTTGAGCGTCAA
SOCS3(R)	CATGTAGTGGTGCACCAGCT
MMP-1(F)	GGCTCCGAGAAATGCAATCT
MMP-1(R)	CCACTTCAGAATGGGACATA
COX-2(F)	TGGTGCCTGGTCTGATGATG
COX-2(R)	GCAATGCGGTTCTGATACTG
IL-10 (F)	GGAGCAGGTGAAGAGTGATT
IL-10 (R)	AGACTCAATACACACTGCAG
IL-2(F)	GGATGCTCACCTTCAAATTT
IL-2(R)	AGATCTTTCAATTCTGTGGC
MCP-1(F)	ATGCTTCTGGGCCTGCTGTT
MCP-1(R)	CAGCTTCTTTGGGACACCTG
IL-1α(F)	TTCCCTCAACCAAACTATAT
IL-1α(R)	ACGGGCTGGTCTTCTCCTTG

Supplemenatry materials and methods

RNA extraction and quantitative real time PCR (qRT-PCR)

Total RNA isolation from RAW264.7 cells was performed using an RNAsimple Total RNA kit (Tiangen, China) as described previously (Wang et al., 2008;Wang et al., 2011). The RNA was reverse transcribed to cDNA using the Strand cDNA Synthesis kit (Thermo, USA). Quantitative real-time PCR was performed using the Power SYBR Green PCR Master Mix protocol (Applied Biosystems, Foster City, CA) with specific primers designed to amplify the genes of IL-1α, IL-1β, IL-2, IL-10, matrix metalloproteinase-1 (MMP-1), macrophage chemoattractant protein-1 (MCP-1), cyclooxygenase-2 (COX-2), lectin-like oxidized LDL receptor-1 (LOX-1), superoxide dismutase 1 (SOD-1) and suppressor of cytokine signaling 3 (SOCS3).

Protein extraction and immunoblot detection of phosphorylated STAT3

The pellets of RAW264.7 cells treated with QCT and LPS were lysed by RIPA buffer containing 1% NP-40, 0.5% deoxycholate, 0.1% SDS (Beyotime, China) in the presence of protease inhibitor and centrifuged at 15,000 × g for 15 min at 4 °C. The supernatant fraction was collected and the protein concentration was determined by the method of bicinchoninic acid (BCA) assay (Beyotime, China). Equal amounts of protein extract (30 μg per well) were separated by 12% SDS poly acrylamide gel electrophoresis (SDS-PAGE). Separated proteins were electrotransferred to a 0.2 μm nitrocellulose membrane and blocked in 5% non-fat milk in 1 × TBST for 1 hour. The blot was incubated with rabbit anti-STAT3 at a dilution of 1:1000 (Cell Signaling, USA) or rabbit anti-pSTAT3 at

a dilution of 1:1000 (Cell Signaling, USA) at 4 °C overnight followed by horseradish peroxidase-conjugated secondary goat anti-rabbit antibody (Gene, China) at 1:2000 dilution. The membrane was then incubated with SuperSignal West Pico Chemiluminent Substrates (Thermo, USA) for 2 min and developed under automatic multifunction chemiluminescent detection system (Tanon, China). The signal was quantified by densitometry using Gel-Pro Analyzer (Media Cybernetics, USA).

Enzyme-Linked Immunosorbent Assay (ELISA)

IL-1β, MCP-1, COX2 and PGE2 concentrations in the total protein of each group were measured using related ELISA kits (Elabscience, China) according to the operating instructions. Briefly, 100μL of standard, blank, or equal amounts of protein extract (50 μg per well) was added to the bottom of micro ELISA plate well and incubated for 90 min at 37°, after removing the liquid of each well, 100 μL of biotinylated detection antibody working solution was added immediately and incubated for 1 hour, then wells were washed three times and 100 μL of HRP conjugate working solution was added and incubated for 30 min, followed by repeat wash process. Subsequently, 90 μL of substrate solution was added and incubated for another 20 min at 37°C, then 50 μL of stop solution was added immediately to each well. Finally, the optical density (OD value) of each well was determined by spectrofluorimetry at 450nm.

MTT assay

To study the toxic effects of QCT on viability of RAW 264.7 cells, cells were seeded into 96-well plates at 8×10^3 cells/well. After incubation with 10, 20, 50, 100, 200 μM QCT for 24 hours, 48 hours, or 72 hours, cells were incubated with 10 μl of 5 mg/ml MTT for 4 h at 37 °C. The medium was aspirated carefully and the resultant MTT formazan was extracted with 100 μl DMSO and quantified by spectrofluorimetry at 450nm.

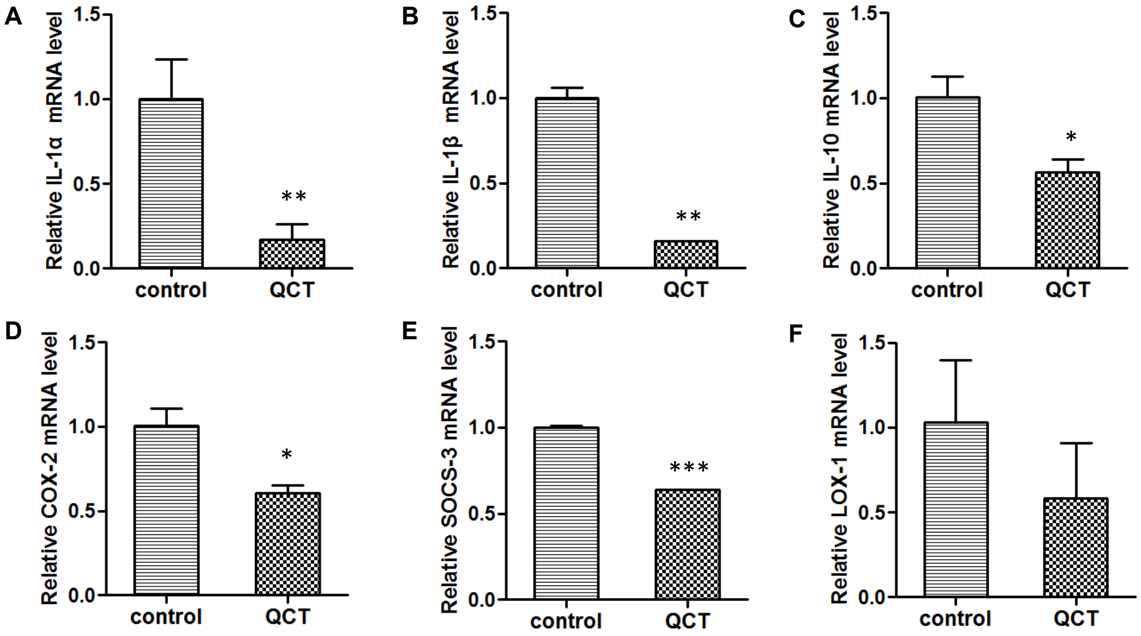

Figure S1. QCT alone decreased mRNA levels of IL-1α, IL-1β, IL-10, COX-2, SOCS3 and LOX-1. QCT (20 μM) treated RAW264.7 cells for 24 hours and then cells were collected for qRT-PCR test. **P* < 0.05, ***P* < 0.01 vs. control group. Data represent the mean value ± S.D. from three separate experiments. The student's t-test was used to calculate *P* values.

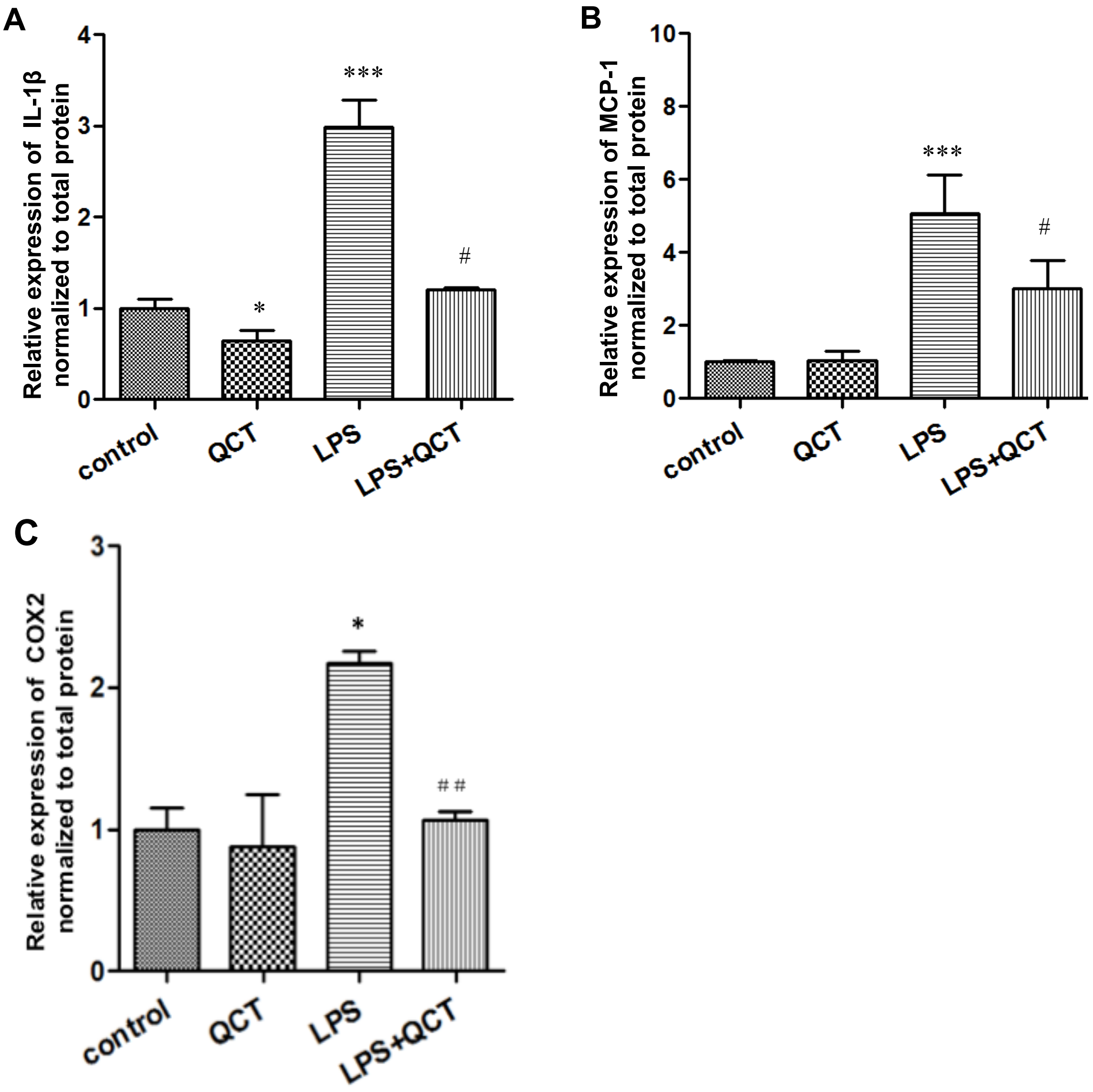

Figure S2. QCT inhibited the protein levels of IL-1β and MCP-1 in LPS-treated RAW264.7 cells. RAW264.7 cells were grown in 6-well plates for 24 hours, control group or QCT only group was always treated with DMSO or QCT, cells in the last two groups were treated with DMSO or 20 μM QCT for 24 hours, and then stimulated with 500ng/mL of LPS for another 6 hours. Total protein in each group was extracted and equal amounts of protein (50 μg) were examined by ELISA. Values for expression levels of IL-1β (**A**), MCP-1 (**B**) and COX-2 (**C**) were set as 100 % of control group. **P* < 0.05, ****P* < 0.001 compared with control group; #*P* < 0.05 compared with LPS group. The bars represent mean ± SD from three independent experiments. One-way ANOVA analysis was used to calculate *P* values.

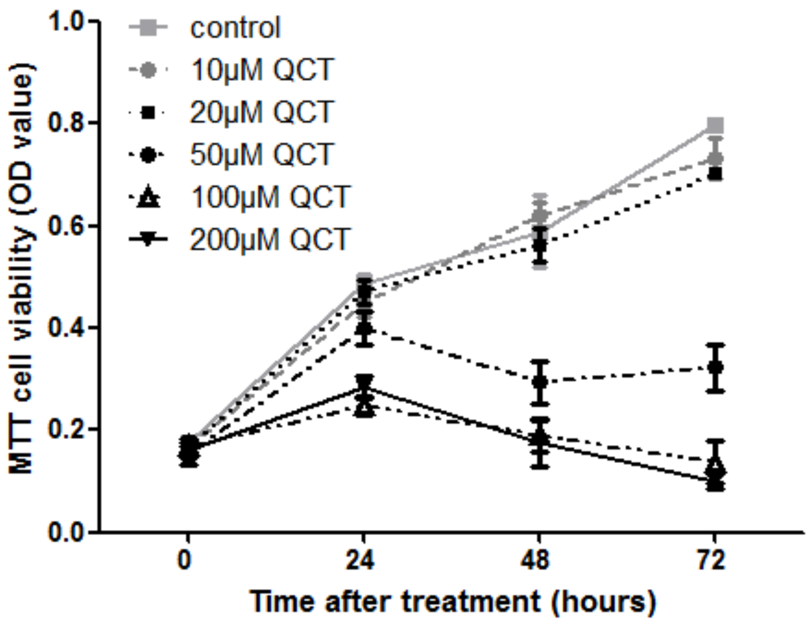

Figure S3. The results of MTT assay. RAW264.7 cells were exposed to various concentrations of QCT for 24 hours, 48 hours, or 72 hours. Cell viability was determined by MTT assay. Data represent the mean value ± S.D. from three separate experiments. One-way ANOVA analysis was used to calculate *P* values.

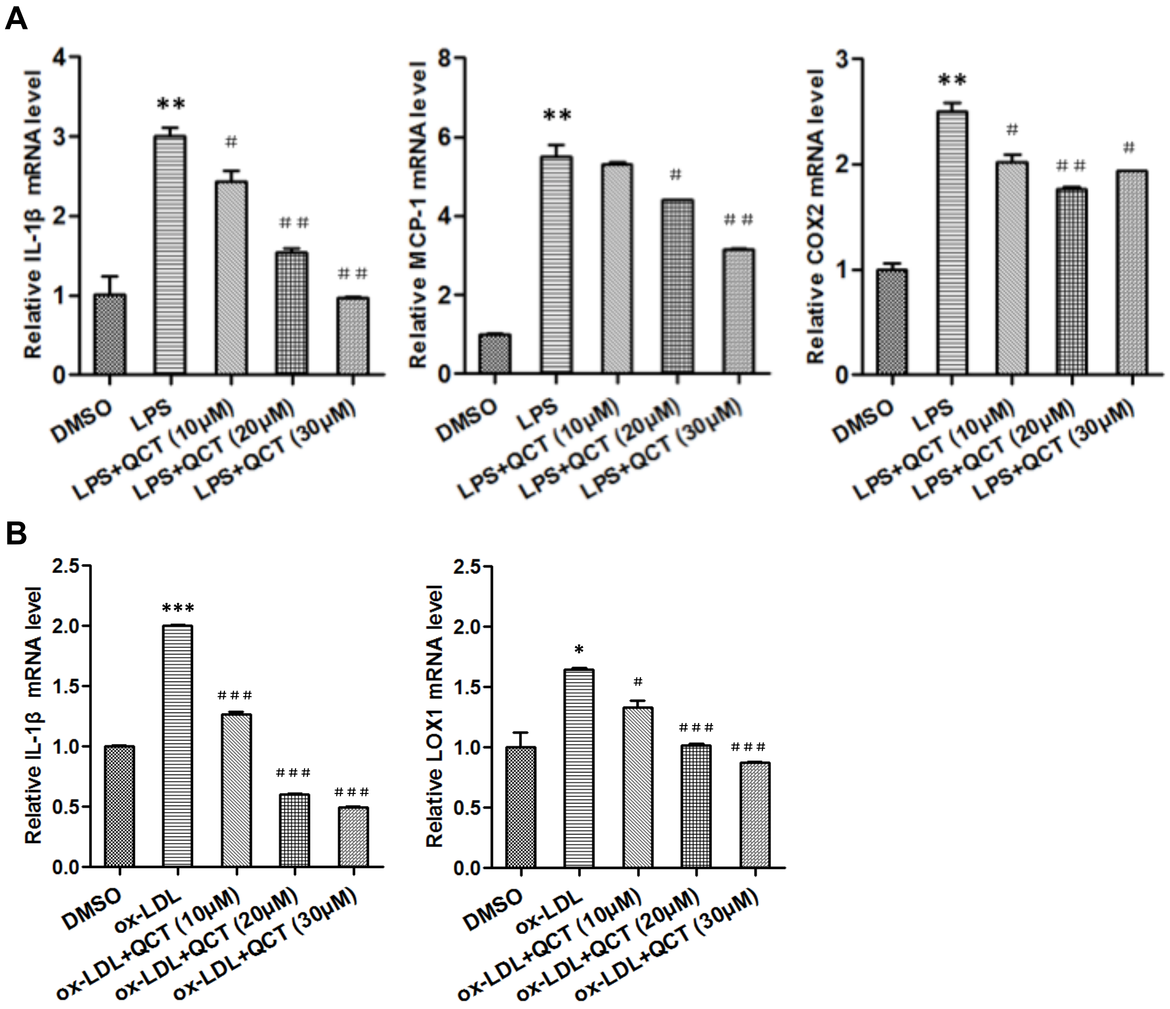

Figure S4. The effects of QCT with different concentrations on cytokine expression triggered by LPS. (A) RAW264.7 cells were pretreated with DMSO, 10 μM, 20 μM or 30 μM QCT for 24 hours, followed by exposure to LPS (500ng/mL) for another 6 hours, cells in control group was always treated with DMSO. At the end of the incubation period, cells were harvested and transcript levels of IL-1β, MCP-1 and COX-2 were examined by qRT-PCR, normalized to the transcript level of 36B4. $^{**}P < 0.01$, $^{***}P < 0.001$ vs. the control group; $\#P < 0.05$, $\#\#P < 0.01$ vs. the LPS treated group. The bars represent mean ± SD from three independent experiments. (**B**) RAW264.7 cells were pretreated with DMSO, 10 μM, 20 μM or 30 μM QCT for 24 hours, followed by exposure to oxLDL (50 μg/mL) for a further 24-hour period., cells in control group was always treated with DMSO. At the end of the incubation period, cells were harvested and transcript levels of IL-1β and LOX-1 were examined by qRT-PCR, normalized to the transcript level of 36B4. $^{*}P < 0.05$, $^{***}P < 0.001$ vs. the control group; $^{\#}P < 0.05$, $^{\#\#\#}P < 0.001$ vs. the oxLDL-stimulated group. The bars represent mean ± SD of three independent experiments.

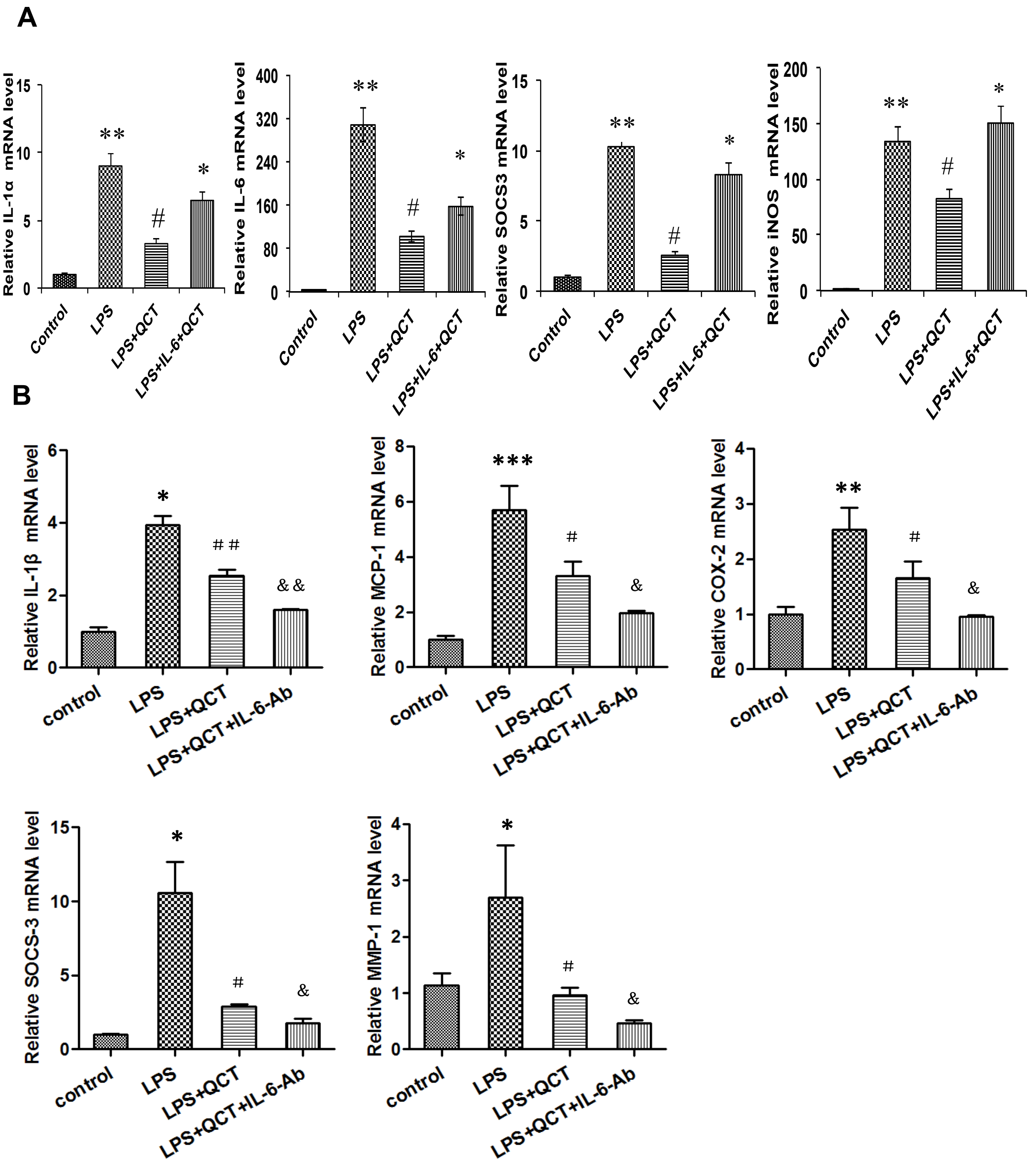

Figure S5. IL-6 and IL-6 antibody affected the suppression of QCT on LPS-induced mRNA levels of proinflammatory genes. (A) QCT (20 μM) treated RAW264.7 cells for 24 hours. Before cells were collected, cells were treated with IL-6 (10ng/mL) for 1 hour or LPS (500ng/mL) for 6 hours for qRT-PCR test. $^{\#}P < 0.05$, $^{**}P < 0.01$ vs. control group. $^{*}P < 0.05$ vs. LPS+QCT group. Data represent the mean value ± S.D. from three separate experiments. (**B**) RAW 264.7 cells were grown in 6-well plates for 24 hours, followed by treated with 20 μM QCT and 20 ng/mL IL-6-Antibody (IL-6-Ab) for 24 hours, and then stimulated with 500ng/ml of LPS for another 6 hours. The cultured cells

were harvested and transcript levels of the following cytokines were examined by qRT-PCR, IL-1β, MCP-1, COX-2, SOCS-3 and MMP-1. **P* < 0.05, ***P* < 0.01, *** *P* < 0.001 vs. control group; #*P* < 0.05, ##*P* < 0.01 vs. LPS-treated group; &*P* < 0.05, &&*P* < 0.01 vs. LPS+QCT-treated group. The bars represent mean ± SD.

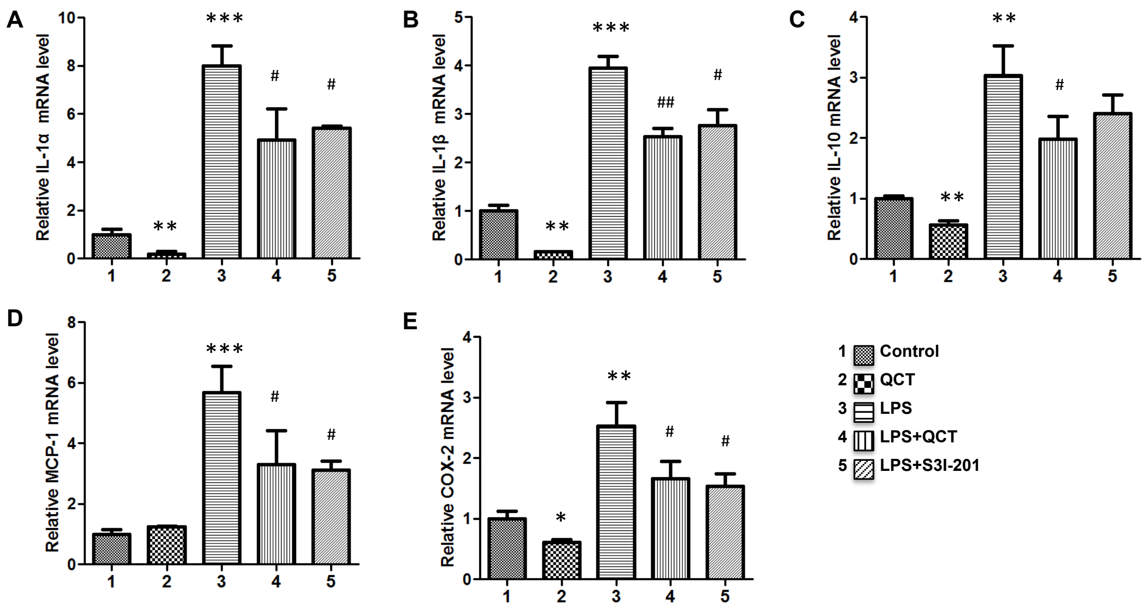

Figure S6. mRNA levels of proinflammatory genes after LPS+QCT or LPS+S3I-201 treatment. Cells in control or QCT treated group was always treated with DMSO or 20 μM QCT, and cells in the last three groups were treated with DMSO, 20 μM QCT or 100 μM S3I-201 for 24 hours, and then stimulated with 500ng/mL of LPS for another 6 hours. The cells were harvested and transcript levels of the following cytokines were examined by qRT-PCR, IL-1α (**A**), IL-1β (**B**), IL-10 (**C**), MCP-1 (**D**) and COX-2 (**E**). *$P < 0.05$, **$P < 0.01$, ***$P < 0.001$ vs. the control group; $^{\#}P < 0.05$, $^{\#\#}P < 0.01$ vs. the LPS treated group. The bars represent mean ± SD from three independent experiments. One-way ANOVA analysis was used to calculate *P* values.

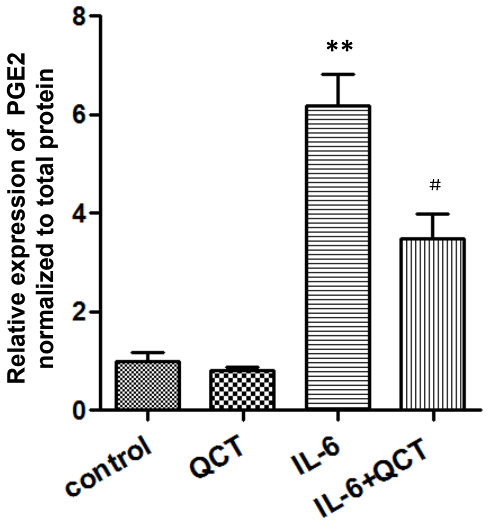

Figure S7. QCT inhibited the expression of PGE2 in IL-6-treated RAW264.7 cells. RAW 264.7 cells were grown in 6-well plates for 24 hours, control group or QCT only group was always treated with DMSO or QCT, cells in the last two groups were treated with DMSO or 20 μM QCT for 24 hours, and then stimulated with 10ng/mL of IL-6 for another 1 hour. Total protein in each group was extracted and equal amounts of protein (50 μg) were examined by ELISA. Values for expression levels of PGE2 were set as 100 % of control group. $^{**}P < 0.01$ compared with control group; $^{\#}P < 0.05$, compared with LPS group. The bars represent mean ± SD from three independent experiments.

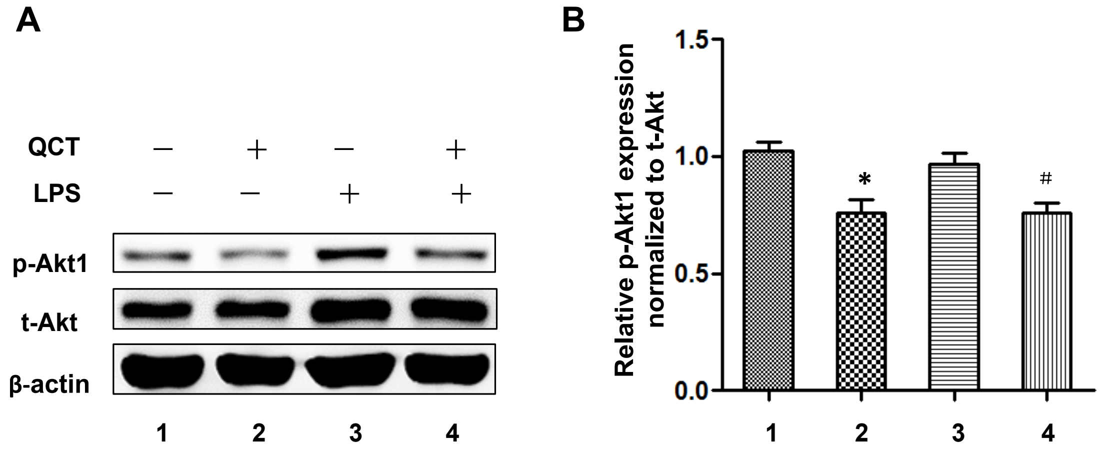

Figure S8. QCT treatment inhibits endogenous and LPS-induced activation of Akt1 in RAW264.7 cells. (**A**) Representative immunoblot showing phosphorylated Akt1 (p-Akt1) and total Akt (t-Akt) protein levels in RAW264.7 cells treated with DMSO, QCT (20 μM), LPS (500ng/ml), LPS (500 ng/ml) + QCT (20 μM) and LPS (500 ng/ml) + S3I-201 (100 μM). β-actin as a loading control. (**B**) Densitometry was used to quantify relative p-Akt1 protein levels normalized to t-Akt1 protein levels $*P < 0.05$ vs. control group; $^{\#}P < 0.05$ vs. LPS-induced group. One-way ANOVA analysis was used to calculate P values. The bars represent mean ± SD. All data represent at least three independent experiments.

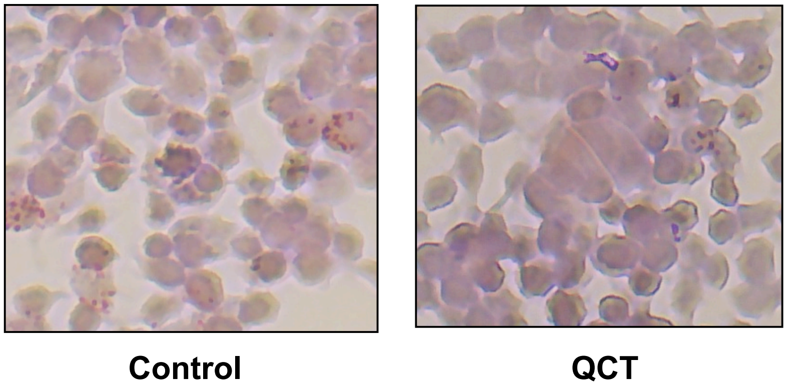

Figure S9. QCT did not cause toxicity to the cells. DMSO (as control) or QCT (20μM) treated RAW264.7 cells for 24 hours and then cells were collected for Oil red O staining.
